# Supplementary material for: Mediators of the association between psychological distress and mortality in people diagnosed with cancer
Source: Nat Commun. 2025 Dec 12;16:11216. doi: 10.1038/s41467-025-66059-2 (PMC12715235; doi:10.1038/s41467-025-66059-2)
Supplement: Supplementary file 1 — Reporting Summary [file 41467_2025_66059_MOESM1_ESM.pdf]

nature portfolio

Corresponding author(s): Natalie Miller

Last updated by author(s): Jun 26, 2026

Reporting Summary

Nature Portfolio wishes to improve the reproducibility of the work that we publish. This form provides structure for consistency and transparency in reporting. For further information on Nature Portfolio policies, see our Editorial Policies and the Editorial Policy Checklist.

Statistics

For all statistical analyses, confirm that the following items are present in the figure legend, table legend, main text, or Methods section.

n/a	Confirmed	
☐	☒	The exact sample size (*n*) for each experimental group/condition, given as a discrete number and unit of measurement
☐	☒	A statement on whether measurements were taken from distinct samples or whether the same sample was measured repeatedly
☐	☒	The statistical test(s) used AND whether they are one- or two-sided<br>*Only common tests should be described solely by name; describe more complex techniques in the Methods section.*
☐	☒	A description of all covariates tested
☐	☒	A description of any assumptions or corrections, such as tests of normality and adjustment for multiple comparisons
☐	☒	A full description of the statistical parameters including central tendency (e.g. means) or other basic estimates (e.g. regression coefficient) AND variation (e.g. standard deviation) or associated estimates of uncertainty (e.g. confidence intervals)
☒	☐	For null hypothesis testing, the test statistic (e.g. F, t, r) with confidence intervals, effect sizes, degrees of freedom and P value noted<br>*Give P values as exact values whenever suitable.*
☒	☐	For Bayesian analysis, information on the choice of priors and Markov chain Monte Carlo settings
☒	☐	For hierarchical and complex designs, identification of the appropriate level for tests and full reporting of outcomes
☒	☐	Estimates of effect sizes (e.g. Cohen's d, Pearson's r), indicating how they were calculated

Our web collection on statistics for biologists contains articles on many of the points above.

Software and code

Policy information about availability of computer code

Data collection	No software was used
Data analysis	We used Stata (Version 17) for UK Biobank analyses, SAS (Version 9.4) for Finnish Public Sector study analyses, and R (Version 4.4.2) for meta-analyses.

For manuscripts utilizing custom algorithms or software that are central to the research but not yet described in published literature, software must be made available to editors and reviewers. We strongly encourage code deposition in a community repository (e.g. GitHub). See the Nature Portfolio guidelines for submitting code & software for further information.

Data

Policy information about availability of data

All manuscripts must include a data availability statement. This statement should provide the following information, where applicable:

- Accession codes, unique identifiers, or web links for publicly available datasets
- A description of any restrictions on data availability
- For clinical datasets or third party data, please ensure that the statement adheres to our policy

Researchers registered with UK Biobank can apply for access to the database by completing an application. This must include a summary of the research plan, data-fields required, any new data or variables that will be generated, and payment to cover the incremental costs of servicing an application (https://

www.ukbiobank.ac.uk/enable-your- research/apply-for-access). In the Finnish Public Sector study, pseudonymised questionnaire data can be shared by request to the investigators. Linked health records require separate permission from the Findata, the Health and Social Data Permit Authority in Finland.

Research involving human participants, their data, or biological material

Policy information about studies with human participants or human data. See also policy information about sex, gender (identity/presentation), and sexual orientation and race, ethnicity and racism.

Reporting on sex and gender	In both UK Biobank and the FPS, sex was recorded at baseline by self-report. No data on gender identity was collected. Sex was included as a covariate in all models. Analyses were not stratified by sex as the aim of the study was to examine overall associations between psychological distress and mortality rather than sex-specific associations.<br><br>We have included the following numbers of male and female participants in the Results: "The UK Biobank sample included 13,349 participants (mean age = 60, SD = 7.1), of whom 6,836 were female (51.2%) and 6,513 were male (48.8%). The FPS sample included 5,739 participants (mean age 57.5, SD 10.2), of whom 4,752 were female (82.8%) and 987 were male (17.2%).
Reporting on race, ethnicity, or other socially relevant groupings	We use ethnicity as a covariate as it is associated with the exposure (distress) and outcome (mortality) of our analyses.
Population characteristics	See below (study design)
Recruitment	UK Biobank is a large prospective cohort study that identified participants via the UK National Health Service (NHS) records. Of the 9.1 million adults eligible for inclusion in UK Biobank, 502,665 adults aged 38-73 years participated in a baseline clinical examination between 2006 and 2010. FPS is a prospective cohort study of public sector personnel in 11 towns and five wellbeing services counties in Finland. FPS includes individuals who responded to surveys during the periods 2000-2002, 2004-2005, 2008-2009, 2012-2013, and 2016–2017.
Ethics oversight	UK Biobank was approved by the National Health Service National Research Ethics Service. The present study was conducted using the UK Biobank Resource (application numbers 60565). FPS was approved by the Helsinki Uusimaa Hospital District Ethics Committee (HUS/1210/2016). All participants provided written informed consent prior to participation in data collection.

Note that full information on the approval of the study protocol must also be provided in the manuscript.

Field-specific reporting

Please select the one below that is the best fit for your research. If you are not sure, read the appropriate sections before making your selection.

☐ Life sciences ☒ Behavioural & social sciences ☐ Ecological, evolutionary & environmental sciences

For a reference copy of the document with all sections, see nature.com/documents/nr-reporting-summary-flat.pdf

Behavioural & social sciences study design

All studies must disclose on these points even when the disclosure is negative.

Study description	This is a prospective, observational, multi-cohort study
Research sample	This prospective, observational, multi-cohort study used data from the UK Biobank for the primary analysis and the Finnish Public Sector study (FPS) for validation. UK Biobank is a large prospective cohort study that identified participants via the UK National Health Service (NHS) records. Of the 9.1 million adults eligible for inclusion in UK Biobank, 502,665 adults aged 38-73 years participated in a baseline clinical examination between 2006 and 2010. FPS is a prospective cohort study of public sector personnel in 11 towns and five wellbeing services counties in Finland. FPS includes individuals who responded to surveys during the periods 2000-2002, 2004-2005, 2008-2009, 2012-2013, and 2016–2017. For the present study, we included participants diagnosed with cancer, with the first diagnosis occurring four years or less before baseline assessments, consistent with previous studies.
Sampling strategy	See above
Data collection	Cancer at baseline<br>Data on cancer type and date of diagnosis were obtained from national cancer registries. Cancer type was coded according to the 9th and 10th revisions of the International Classification of Diseases (ICD-9 and ICD-10) (Supplementary file). In FPS, data on cancer stage were also obtained from national registries.<br><br>Psychological distress<br>In UK Biobank, psychological distress was measured at baseline using the 4-item version of the Patient Health Questionnaire (PHQ-4).44 The PHQ-4 asks participants about the frequency of low mood, anhedonia, feelings of anxiety and inability to control worrying over the past two weeks. Responses are rated on a four-point Likert scale from 0 "not at all" to 3 "nearly every day". The total score ranges from 0-12, with higher scores indicating greater distress. The established cut-off score of 6 or more was used to

denote psychological distress.45,46 The PHQ-4 has been shown to be a valid measure of psychological distress in people diagnosed with cancer.47,48

In FPS, distress was measured at baseline using the 12-item General Health Questionnaire (GHQ-12),49 which assesses the severity of a mental distress over the past few weeks. Items are scored on a 4-point Likert scale (from 0-3), with higher scores indicating worse mental health. The total score ranges from 0-36. Based on the optimal cut-off point of 3 established in a Finnish validation study,50 participants were categorised as non-distressed (GHQ scores 0-3) and distressed (GHQ scores 4-12). The GHQ-12 has been shown to be a valid measure of psychological distress in people LWBC.51,52

Mediators
Mediators assessed at baseline included alcohol consumption, physical activity, smoking, body mass index (BMI), sleep duration, and sleep quality were assessed in both cohorts, while data on inflammation (high-sensitivity C-reactive protein concentration in serum) and dietary intake (fruit, vegetable, red meat, and processed meat) were measured only in UK Biobank. Table 1 summarises the mediators assessed in UK Biobank and FPS, along with their methods of ascertainment. In addition, Supplementary File 1 provides full detail of how all mediators were assessed.

Covariates
Covariates included self-reported age, sex, ethnicity (dichotomised into White/non-White), education (low/intermediate/high), number of comorbidities, cancer stage (FPS only), age at cancer diagnosis, time between diagnosis and baseline assessments, and antidepressant use (yes/no). Comorbidities were assessed using hospitalisation data coded according to the ICD-10, including diabetes, Alzheimer's disease, asthma, dementia, Parkinson's disease, hypertension, angina, heart attack, stroke, heart failure, heart murmur, abnormal heart rhythm, and chronic kidney disease, categorised as 0 versus 1+. Date of diagnosis was used to determine age at cancer diagnosis and time between diagnosis and baseline assessments. In UK Biobank, antidepressant use was assessed through self-reported medications (field 20003). A list of antidepressants developed in prior UK Biobank studies was used to derive a variable indicating if participants had taken at least one antidepressant medication.53 In FPS, antidepressant use was determined from The Medication Register/Social Insurance Institution of Finland (Anatomical Therapeutic Chemical code N06A), indicating if participants had purchased an antidepressant medication or not.

Timing

UK Biobank: 2006-2021
FPS: 2000-2018

Data exclusions

No data were excluded from analysis

Non-participation

UK Biobank had an initial response rate of 5.5%.
FPS had an initial response rate of 60-70%

Randomization

Participants were not allocated into groups

Reporting for specific materials, systems and methods

We require information from authors about some types of materials, experimental systems and methods used in many studies. Here, indicate whether each material, system or method listed is relevant to your study. If you are not sure if a list item applies to your research, read the appropriate section before selecting a response.

Materials & experimental systems

n/a	Involved in the study	
☒	☐	Antibodies
☒	☐	Eukaryotic cell lines
☒	☐	Palaeontology and archaeology
☒	☐	Animals and other organisms
☒	☐	Clinical data
☒	☐	Dual use research of concern
☒	☐	Plants

Methods

n/a	Involved in the study	
☒	☐	ChIP-seq
☒	☐	Flow cytometry
☒	☐	MRI-based neuroimaging

Plants

Seed stocks	N/A
Novel plant genotypes	N/A
Authentication	N/A
