# Supplementary material for: Mediators of the association between psychological distress and mortality in people diagnosed with cancer
Source: Nat Commun. 2025 Dec 12;16:11216. doi: 10.1038/s41467-025-66059-2 (PMC12715235; doi:10.1038/s41467-025-66059-2)
Supplement: Supplementary file 2 — Description of Additional Supplementary Files [file 41467_2025_66059_MOESM2_ESM.pdf]

Description of Additional Supplementary Files

Supplementary Dataset 1 Legend: Characteristics of individuals diagnosed with cancer within 4 years prior to baseline assessments in the UK Biobank and Replication Cohort.
Notes. SD = standard deviation.
Values are presented as means (SD) for continuous variables and n (%) for categorical variables.
a Pooled ns and %s are shown across the 20 imputed datasets.

Supplementary Dataset 2 Legend: UK Biobank participant characteristics in observed and imputed data (*N* = 13,349).
Notes. SD = standard deviation.
Values are presented as means (SD) for continuous variables and n (%) for categorical variables.
a For imputed data, pooled ns and %s are shown across the 20 imputed datasets.

Supplementary Dataset 3 Legend: Association between psychological distress and all-cause mortality risk among people LWBC.
Notes. Cox proportional hazards models (two-tailed).
*p<0.05; **p<0.01, ***p<0.001.
CI = 95% confidence interval, HR = hazard ratio.
Reference category = low distress.
Model 1 adjusted for age and sex.
Model 2 adjusted for age, sex, ethnicity, education, number of comorbidities, age at cancer diagnosis, time between cancer diagnosis and depressive symptoms assessment, and antidepressant medication (yes/no).

Supplementary Dataset 4 Legend: Association between psychological distress and cancer-specific mortality risk among people LWBC.
Notes. Competing risk regression (two-tailed).
*p<0.05; **p<0.01, ***p<0.001.
CI = 95% confidence interval, SHR = sub-distribution hazard ratio.
Reference category = low distress.
Model 1 adjusted for age and sex.
Model 2 adjusted for age, sex, ethnicity, education, number of comorbidities, age at cancer diagnosis, time between cancer diagnosis and depressive symptoms assessment, and antidepressant medication (yes/no).
