# Supplementary material for: Mediators of the association between psychological distress and mortality in people diagnosed with cancer
Source: Nat Commun. 2025 Dec 12;16:11216. doi: 10.1038/s41467-025-66059-2 (PMC12715235; doi:10.1038/s41467-025-66059-2)
Supplement: Supplementary file 8 — Supplementary File [file 41467_2025_66059_MOESM8_ESM.docx]

**Supplementary File**

**Cancer ICD codes**

The cancer type variable was collapsed into the following categories: head and neck (ICD-9: 140-149; ICD-10: C00-C14); gastrointestinal excluding colorectal (ICD-9: 150-152; ICD-10: C15-C17, C22-C26); colorectal (ICD-9: 154; ICD-10: C18); respiratory including lung (ICD-9: 160-165; ICD-10 C30-C39); bone, mesothelial and soft tissue (ICD-9: 158, 170-171, 176; ICD-10: C40-C41, C45-C49); melanoma (ICD-9: 172; ICD-10: C43); non-melanoma skin (ICD-9 173; ICD-10: C42); breast (ICD-9: 174-175; ICD-10: C50); female reproductive (ICD-9: 179-184; ICD-10: C51-C58); male reproductive (ICD-9: 185-187; ICD-10: C60-C63); urinary tract (ICD-9: 188-189; ICD-10: C64-C68); endocrine (ICD-9: 193-194; ICD-10: C74-C79); lymphoma (ICD-9: 200-202; ICD-10: C81-C86, C96); multiple myeloma (ICD-9: 203; ICD-10: C88-C90); leukaemia (ICD-9: 204-208; ICD-10: C91-C95); and unspecified or other (ICD-9: 45-47, 50, 55, 59, 209, 235-238; ICD-10: C69-C73, C80, C97, D37-48).

**Measurement of mediators in UK Biobank**

All mediators were assessed at baseline.

**Diet and alcohol consumption**

Dietary behaviours were assessed using a touchscreen questionnaire. The touchscreen questionnaire included 29 questions on diet and 18 questions on alcohol. The questions on diet asked about the frequency of consumption over the past year of different food groups.

**Fruit intake**

Participants reported the number of pieces of fresh fruit eaten per day (field 1309). Participants also reported the number of pieces of dried fruit eaten per day (field 1319). The response option less than one (value = -10) was coded as half a piece. One piece of fresh fruit counted as a serving and two pieces of dried fruit counted as a serving. The total number of servings of fruit per day was calculated and dichotomised into <2 vs. ≥2 servings a day.

**Vegetable intake**

Participants reported the number of tablespoons of raw vegetables eaten per day (field 1299), and the number of tablespoons of cooked vegetables eaten per day (field 1289). The response option less than one (value = -10) was coded as half a tablespoon. Three tablespoons were considered as one portion of raw/cooked vegetables. The total number of portions of vegetables per day was calculated and dichotomised into <2 vs. ≥2 servings a day.

**Red meat intake**

Participants reported the frequency of consumption of beef (field 1369), lamb/mutton (field 1379) and pork (field 1389). The frequencies of consumption for beef, lamb/mutton and pork intake were summed using the following coding: ‘never’ = 0, ‘less than once a week’ = 0.5, ‘once a week’ = 1, ‘2-4 times a week’ = 3, ‘5-6 times a week’ = 5.5 and ‘once or more daily’ = 7. This variable was then dichotomised into <3 vs ≥3 times per week, given the WCRF recommends that people LWBC limit consumption of red meat to less than three servings a week [29].

**Processed meat intake**

Participants reported the frequency of processed meat consumption (field 1349). Responses were coded as follows: ‘never’ = 0, ‘less than once a week’ = 0.5, ‘once a week’ = 1, ‘2-4 times a week’ = 3, ‘5-6 times a week’ = 5.5 and ‘once or more daily’ = 7. This variable was then dichotomised into never or rarely vs. at least once a week, given the WCRF recommends that people LWBC avoid processed meat [29]. .

**Alcohol consumption**

Participants reported their current drinking status (daily/three to four times a week/once or twice a week/one to three times a month/special occasions only/never) (field 1558), as well as their average weekly consumption of the following drinks: red wine (field 1568), white wine and champagne (field 1578), beer and cider (field 1588), and fortified wine (data 1608). All these variables were used to derive a single variable indicating the average number of units of alcohol consumed per week. Units of alcohol were defined as follows: a standard glass of wine (175ml) = 2.1 units; a pint or can of beer/lager/cider = 2.4 units; and a 25ml single shot of spirits = 1 unit. The total number of units of alcohol consumed per week was calculated as the sum of weekly units in all the categories. Participants who reported drinking one to three times a month, on special occasions only, or never on the current drinking status question were coded as having an average weekly alcohol consumption of 0 units. This variable was then dichotomised into <14 vs. ≥14 weekly units, in line with UK national guidelines [30].

**Physical activity**

Physical activity was assessed using a modified version of the International Physical Activity Questionnaire (IPAQ) comprising six questions on frequency (fields 864, 884, and 904) and duration (fields 874, 894, and 914) of walking, moderate activity and vigorous activity ) [28]. The response ‘unable to walk’ in 864 was recoded to 0. The total number of MET minutes per week for walking, moderate activity and vigorous activity was calculated by multiplying the frequency and duration of physical activity in each category by 3.3 for walking, 4.0 for moderate activity, and 8 for vigorous activity. The total number of MET minutes per week for all activity combined was calculated by summing MET minutes per week in each category. This variable was dichotomised into <600 (physically inactive) vs ≥600 (physically active) MET minutes per week, in line with the IPAQ scoring manual [31].

**Smoking status**

Smoking status (current/previous/never) was assessed using a single item and collapsed into two categories: current smoker/non-smoker (field 20116).

**Sleep duration**

Sleep duration was self-reported (field 1160) and categorised into short (<7 hours) or long sleep (>9 hours) vs. normal sleep (7-9 hours).

**Sleep quality**

A sleep quality score was calculated using five different sleep behaviours: sleep duration (field 1160), chronotype (field 1180), insomnia (field 1200), snoring (field 1210) and daytime sleepiness (field 1220), using a method reported by several other studies [32, 33]. Each behaviour was categorised into low or high risk. Low risk behaviours included early chronotype (‘morning’ or ‘more morning than evening’), sleep duration of 7-9 hours per day, reported never or rarely insomnia symptoms, no snoring, and no frequent daytime sleepiness (‘never/rarely’ or ‘sometimes’). Low risk behaviours were each given a score of 1 and high-risk behaviours were each given a score of 0. A total sleep quality score was calculated by summing the scores for each of the five behaviours. The total sleep quality score ranged from 0-5 and was collapsed into the following categories: healthy sleep quality (scores 4-5) vs moderate or poor sleep quality (scores 0-3).

**Inflammation**

Blood samples were collected at baseline. Serum C-reactive protein (CRP) levels (field 30710) were measured by immunoturbidimetric high-sensitivity analysis on a Beckman Coulter AU5800. The minimum detection limit was 0·08 mg/L. A cut-off of 3mg/l or more was used to denote high inflammation.

**Body mass index**

Height and weight were measured at baseline and body mass index (BMI) was calculated as weight(kg)/(height(m))^2^ (field 21001). BMI was dichotomised into underweight or healthy (<25kg/m^2^) vs. overweight or obese (≥25kg/m^2^).

**Measurement of mediators in Finnish Public Sector study**

All mediators were assessed at baseline.

**Physical activity**

Assessed using questions on average weekly hours of both leisure time and commuting physical activity using four different intensity levels: walking, brisk walking, jogging and running (or other activities with a corresponding intensity level). The time spent on activity at each intensity level in hours per week was multiplied by the average energy expenditure of each activity, expressed in metabolic equivalent (MET).Dichotomised into less than 600 MET-min/week vs. 600 or more MET-min/week.

**Alcohol consumption**

Amount of beer, wine and spirits (average units/week). Dichotomised into less than 14 units vs. 14 units or more.

**Smoking status**

Current smoking (yes/no).

**Body Mass Index**

Used self-reported height and weight to measure body mass index (kg/m2). Dichotomised into less than 25 vs. 25 or more.

**Sleep quality**

Sleep difficulties were measured with the Jenkins Sleep Problem Scale including four items: difficulties falling asleep, difficulties maintaining sleep during the night, waking up too early in the morning, and nonrestorative sleep. Participants were asked to estimate how often each of these difficulties had occurred during the previous 4 weeks (never, one–three nights per month, one night per week, two–four nights per week, five–six nights per week, and nearly every night). Any sleep difficulty was created based on the most frequent symptom the participant reported. Dichotomised into less than 2 nights per week vs. 2 or more nights per week.

**Sleep duration**

Measured by asking participants to estimate how many hours they usually sleep per 24 hours. Dichotomised into 7-9 hours vs. <7 or >9 hours.

**Supplementary table 1.** Association between psychological distress (continuous scores) and all-cause mortality risk over 15 years of follow-up among people LWBC (UK Biobank).

|  | All-cause mortality | |
| --- | --- | --- |
|  | HR (95% CI) | p value |
|  | N deaths /N total = 2421/13349 | |
| Model 1 | 1.11 (1.09-1.13) | <0.001*** |
| Model 2 | 1.09 (1.07-1.11) | <0.001*** |

Notes. Cox proportional hazards models (two-tailed).

*p<0.05; **p<0.01, ***p<0.001.

CI = 95% confidence interval, HR = hazard ratio.

Model 1 adjusted for age and sex.

Model 2 adjusted for age, sex, ethnicity, education, number of comorbidities, age at cancer diagnosis, time between cancer diagnosis and depressive symptoms assessment, and antidepressant medication (yes/no).

**Supplementary table 2.** Association between psychological distress (continuous scores) and cancer-specific mortality risk over 15 years of follow-up among people LWBC (UK Biobank).

|  | Cancer-specific mortality | |
| --- | --- | --- |
|  | SHR (95% CI) | p value |
|  | N deaths/N total = 1790/13349 | |
| Model 1 | 1.09 (1.07-1.11) | <0.001*** |
| Model 2 | 1.08 (1.06-1.10) | <0.001*** |

Notes. Competing risk regression (two-tailed).

*p<0.05; **p<0.01, ***p<0.001.

CI = 95% confidence interval, SHR = sub-distribution hazard ratio.

Model 1 adjusted for age and sex.

Model 2 adjusted for age, sex, ethnicity, education, number of comorbidities, age at cancer diagnosis, time between cancer diagnosis and depressive symptoms assessment, and antidepressant medication (yes/no).

**Supplementary table 3.** Association between psychological distress and all-cause mortality risk over 15 years of follow-up among people LWBC, excluding people who died within a year of baseline assessments (UK Biobank).

|  | All-cause mortality | |
| --- | --- | --- |
|  | HR (95% CI) | p value |
|  | N deaths /N total = 2229/13157 | |
| Model 1 | 1.69 (1.44-1.97) | <0.001*** |
| Model 2 | 1.43 (1.22-1.68) | <0.001*** |

Notes. Cox proportional hazards models (two-tailed).

*p<0.05; **p<0.01, ***p<0.001.

CI = 95% confidence interval, HR = hazard ratio.

Model 1 adjusted for age and sex.

Model 2 adjusted for age, sex, ethnicity, education, number of comorbidities, age at cancer diagnosis, time between cancer diagnosis and depressive symptoms assessment, and antidepressant medication (yes/no).

**Supplementary table 4.** Association between psychological distress and cancer-specific mortality risk over 15 years of follow-up among people LWBC, excluding people who died within a year of baseline assessments (UK Biobank).

|  | Cancer-specific mortality | |
| --- | --- | --- |
|  | SHR (95% CI) | p value |
|  | N deaths/N total = 1619/13157 | |
| Model 1 | 1.58 (1.31-1.89) | <0.001*** |
| Model 2 | 1.44 (1.19-1.74) | <0.001*** |

Notes. Competing risk regression (two-tailed).

*p<0.05; **p<0.01, ***p<0.001.

CI = 95% confidence interval, SHR = sub-distribution hazard ratio.

Model 1 adjusted for age and sex.

Model 2 adjusted for age, sex, ethnicity, education, number of comorbidities, age at cancer diagnosis, time between cancer diagnosis and depressive symptoms assessment, and antidepressant medication (yes/no).

**Supplementary table 5.** Association between psychological distress and all-cause mortality risk over 15 years of follow-up among people LWBC, with self-reported comorbidities as covariate (UK Biobank).

|  | All-cause mortality | |
| --- | --- | --- |
|  | HR (95% CI) | p value |
|  | N deaths/N total = 2421/13349 | |
| Model 1 | 1.69 (1.45-1.96) | <0.001*** |
| Model 2 | 1.47 (1.25-1.73) | <0.001*** |

Notes. Cox proportional hazards models (two-tailed).

*p<0.05; **p<0.01, ***p<0.001.

CI = 95% confidence interval, HR = hazard ratio.

Model 1 adjusted for age and sex.

Model 2 adjusted for age, sex, ethnicity, education, number of comorbidities, age at cancer diagnosis, time between cancer diagnosis and depressive symptoms assessment, and antidepressant medication (yes/no).

**Supplementary table 6.** Association between psychological distress and cancer-specific mortality risk over 15 years of follow-up among people LWBC, with self-reported comorbidities as covariate (UK Biobank).

|  | Cancer-specific mortality | |
| --- | --- | --- |
|  | SHR (95% CI) | p value |
|  | N deaths/N total = 1790/13349 | |
| Model 1 | 1.59 (1.34-1.89) | <0.001*** |
| Model 2 | 1.44 (1.19-1.75) | <0.001*** |

Notes. Competing risk regression (two-tailed).

*p<0.05; **p<0.01, ***p<0.001.

CI = 95% confidence interval, SHR = sub-distribution hazard ratio.

Model 1 adjusted for age and sex.

Model 2 adjusted for age, sex, ethnicity, education, number of comorbidities, age at cancer diagnosis, time between cancer diagnosis and depressive symptoms assessment, and antidepressant medication (yes/no).

**Supplementary table 7.** Association between psychological distress and all-cause mortality risk over 19 years of follow-up among people LWBC, using time from diagnosis as the underlying time scale (UK Biobank).

|  | All-cause mortality | |
| --- | --- | --- |
|  | HR (95% CI) | p value |
|  | N deaths /N total = 2421/13349 | |
| Model 1 | 1.69 (1.46, 1.96) | <0.001*** |
| Model 2 | 1.43 (1.23, 1.67) | <0.001*** |

Notes. Cox proportional hazards models (two-tailed).

*p<0.05; **p<0.01, ***p<0.001.

CI = 95% confidence interval, HR = hazard ratio.

Model 1 adjusted for age and sex.

Model 2 adjusted for age, sex, ethnicity, education, number of comorbidities, age at cancer diagnosis, time between cancer diagnosis and depressive symptoms assessment, and antidepressant medication (yes/no).

**Supplementary table 8.** Association between psychological distress and cancer-specific mortality risk over 19 years of follow-up among people LWBC, using time from diagnosis as the underlying time scale (UK Biobank).

|  | Cancer-specific mortality | |
| --- | --- | --- |
|  | SHR (95% CI) | p value |
|  | N deaths/N total = 1790/13349 | |
| Model 1 | 1.59 (1.34, 1.89) | <0.001*** |
| Model 2 | 1.44 (1.20, 1.73) | <0.001*** |

Notes. Competing risk regression (two-tailed).

*p<0.05; **p<0.01, ***p<0.001.

CI = 95% confidence interval, SHR = sub-distribution hazard ratio.

Model 1 adjusted for age and sex.

Model 2 adjusted for age, sex, ethnicity, education, number of comorbidities, age at cancer diagnosis, time between cancer diagnosis and depressive symptoms assessment, and antidepressant medication (yes/no).

**Supplementary table 9.** Association between psychological distress and all-cause mortality risk over 15 years of follow-up among people LWBC, excluding people with non-melanoma skin cancer (UK Biobank).

|  | All-cause mortality | |
| --- | --- | --- |
|  | HR (95% CI) | p value |
|  | N deaths/N total = 2029/9399 | |
| Model 1 | 1.50 (1.28-1.77) | <0.001*** |
| Model 2 | 1.30 (1.10-1.54) | 0.002** |

Notes. Cox proportional hazards models (two-tailed).

*p<0.05; **p<0.01, ***p<0.001.

CI = 95% confidence interval, HR = hazard ratio.

Model 1 adjusted for age and sex.

Model 2 adjusted for age, sex, ethnicity, education, number of comorbidities, age at cancer diagnosis, time between cancer diagnosis and depressive symptoms assessment, and antidepressant medication (yes/no).

**Supplementary table 10.** Association between psychological distress and cancer-specific mortality risk over 15 years of follow-up among people LWBC, excluding people with non-melanoma skin cancer (UK Biobank).

|  | Cancer-specific mortality | |
| --- | --- | --- |
|  | SHR (95% CI) | p value |
|  | N deaths/N total = 1589/9399 | |
| Model 1 | 1.45 (1.21-1.74) | <0.001*** |
| Model 2 | 1.35 (1.12-1.63) | 0.002** |

Notes. Competing risk regression (two-tailed).

*p<0.05; **p<0.01, ***p<0.001.

CI = 95% confidence interval, SHR = sub-distribution hazard ratio.

Model 1 adjusted for age and sex.

Model 2 adjusted for age, sex, ethnicity, education, number of comorbidities, age at cancer diagnosis, time between cancer diagnosis and depressive symptoms assessment, and antidepressant medication (yes/no).

**Supplementary table 11.** Association between psychological distress and all-cause mortality risk over 15 years of follow-up among people LWBC (complete case analysis) (UK Biobank).

|  | All-cause mortality | |
| --- | --- | --- |
|  | HR (95% CI) | p value |
|  | N deaths/N total = 1220/7374 | |
| Model 1 | 1.81 (1.44-2.29) | <0.001*** |
| Model 2 | 1.55 (1.22-1.97) | <0.001*** |

Notes. Cox proportional hazards models (two-tailed).

*p<0.05; **p<0.01, ***p<0.001.

CI = 95% confidence interval, HR = hazard ratio.

Model 1 adjusted for age and sex.

Model 2 adjusted for age, sex, ethnicity, education, number of comorbidities, age at cancer diagnosis, time between cancer diagnosis and depressive symptoms assessment, and antidepressant medication (yes/no).

**Supplementary table 12.** Association between psychological distress and cancer-specific mortality risk over 15 years of follow-up among people LWBC (complete case analysis) (UK Biobank).

|  | Cancer-specific mortality | |
| --- | --- | --- |
|  | SHR (95% CI) | p value |
|  | N deaths/N total = 906/7374 | |
| Model 1 | 1.48 (1.11-1.98) | 0.007** |
| Model 2 | 1.34 (0.99-1.82) | 0.059 |

Notes. Competing risk regression (two-tailed).

*p<0.05; **p<0.01, ***p<0.001.

CI = 95% confidence interval, SHR = sub-distribution hazard ratio.

Model 1 adjusted for age and sex.

Model 2 adjusted for age, sex, ethnicity, education, number of comorbidities, age at cancer diagnosis, time between cancer diagnosis and depressive symptoms assessment, and antidepressant medication (yes/no).

**Supplementary table 13.** Association between psychological distress and all-cause mortality risk, excluding individuals with advanced stage cancer (FPS).

|  | All-cause mortality | |
| --- | --- | --- |
|  | HR (95% CI) | p value |
|  | N deaths/N total = 505/5739 | |
| Model 1 | 1.80 (1.50-2.16) | <0.001*** |
| Model 2 | 1.72 (1.44-2.07) | <0.001*** |
|  | N deaths/N total = 326/5112 | |
| Model 2 (excluded stages 3-6) | 1.56 (1.24, 1.97) | 0.0002** |
|  | N deaths/N total = 213/4291 | |
| Model 2 (excluded stages 2-6) | 1.71 (1.28, 2.28) | 0.0002** |

Notes. Cox proportional hazards models (two-tailed).

*p<0.05; **p<0.01, ***p<0.001.

CI = 95% confidence interval, HR = hazard ratio.

Model 1 adjusted for age and sex.

Model 2 adjusted for age, sex, ethnicity, education, number of comorbidities, age at cancer diagnosis, time between cancer diagnosis and depressive symptoms assessment, and antidepressant medication (yes/no).

**Supplementary table 14.** Association between psychological distress and cancer mortality risk, excluding individuals with advanced stage cancer (FPS).

|  | Cancer mortality | |
| --- | --- | --- |
|  | SHR (95% CI) | p value |
|  | N deaths/N total = 440/5739 | |
| Model 1 | 1.70 (1.47, 2.18) | <0.001*** |
| Model 2 | 1.72 (1.41, 2.10) | <0.001*** |
|  | N deaths/N total = 269/5112 | |
| Model 2 (excluded stages 3-6) | 1.57 (1.21, 2.03) | 0.0007** |
|  | N deaths/N total = 169/4291 | |
| Model 2 (excluded stages 2-6) | 1.76 (1.28, 2.43) | 0.0006** |

Notes. Competing risk regression (two-tailed).

*p<0.05; **p<0.01, ***p<0.001.

CI = 95% confidence interval, HR = hazard ratio.

Model 1 adjusted for age and sex.

Model 2 adjusted for age, sex, ethnicity, education, number of comorbidities, age at cancer diagnosis, time between cancer diagnosis and depressive symptoms assessment, and antidepressant medication (yes/no).
